# CD44 Upregulation in E-Cadherin-Negative Esophageal Cancers Results in Cell Invasion

**DOI:** 10.1371/journal.pone.0027063

**Published:** 2011-11-01

**Authors:** Grégoire F. Le Bras, Gillian L. Allison, Nicole F. Richards, Shazia S. Ansari, M. Kay Washington, Claudia D. Andl

**Affiliations:** 1 Department of Surgery, Vanderbilt Ingram Cancer Center, Vanderbilt University, Nashville, Tennessee, United States of America; 2 Departments of Pathology, Vanderbilt Ingram Cancer Center, Vanderbilt University, Nashville, Tennessee, United States of America; 3 Vanderbilt Ingram Cancer Center, Vanderbilt University, Nashville, Tennessee, United States of America; 4 Department of Cancer Biology, Vanderbilt Ingram Cancer Center, Vanderbilt University, Nashville, Tennessee, United States of America; Emory University, United States of America

## Abstract

E-cadherin is frequently lost during epithelial-mesenchymal transition and the progression of epithelial tumorigenesis. We found a marker of epithelial-mesenchymal transition, CD44, upregulated in response to functional loss of E-cadherin in esophageal cell lines and cancer. Loss of E-cadherin expression correlates with increased expression of CD44 standard isoform. Using an organotypic reconstruct model, we show increased CD44 expression in areas of cell invasion is associated with MMP-9 at the leading edge. Moreover, Activin A increases cell invasion through CD44 upregulation after E-cadherin loss. Taken together, our results provide functional evidence of CD44 upregulation in esophageal cancer invasion.

## Introduction

CD44, or Indian blood group, is a transmembrane glycoprotein and a receptor for hyaluronic acid (HA), a major component of the extracellular matrix [1]. CD44 plays an important role in tissue integrity and is involved in multiple functions associated with cancer progression such as cell migration [2], resistance to apoptosis [3] and presentation of growth factors and proteases [4], [5]. In addition, CD44 has been identified as a specific marker of cancer stem cells [6], [7] and of tumor cells undergoing an epithelial-mesenchymal transition (EMT)-like process [8]. Different isoforms are translated due to alternative splicing of exons 6–15 [9]. The standard form of CD44 (CD44s) is thought to be ubiquitously expressed, while some variant forms, CD44v, have been associated with metastasis [10].

A major step of EMT is the loss of E-cadherin. E-cadherin is a key component of adherens junctions and mediates cell adhesion by interacting with the cytoplasmic proteins β-catenin [11] and p120 [12]. Adherens junctions are connected to the actin cytoskeleton through cytoplasmic linkers such as α-catenin [13]. E-cadherin can be transcriptionally repressed through Snail1, Snail2, Twist, Zeb1 and Zeb2, which can be regulated by TGFβ [14]. Stimulation of breast cancer cells with TGFβ results in co-localization of the matrix metalloproteinase MT1-MMP and CD44 at the cell membrane and induces CD44 cleavage resulting in high levels of soluble CD44 [15]. Conversely, the interaction between hyaluronan and CD44 modulates TGFβ receptor trafficking, which can regulate TGFβ signaling [16].

During EMT, epithelial cells express markers such as CD44, which are known to be expressed in cancer stem cells [8]. Activin A has been shown to be a key regulator of stemness [17]. Activin A, a member of the TGFβ superfamily, has also been described to affect development, hematopoiesis and tumorigenesis [18]–[24]. We therefore wanted to determine whether Activin A induces EMT in esophageal cells and thus links the induction of cell invasion to Activin A and CD44 expression.

We have previously shown the coordinated loss of E-cadherin and TGFβ-receptor II (TGFβRII) in esophageal carcinoma [25]. Other reports have indicated the importance of CD44 in esophageal squamous carcinoma demonstrating a higher expression of CD44v6 [26], [27], but its impact on tumor invasion remains controversial. Therefore, we aimed to study CD44 expression in esophageal squamous epithelium *in vivo* and *in vitro*. In the present study we show that CD44 upregulation is associated with E-cadherin loss in primary esophageal tumor tissues and cancer cell lines. Functionally, we demonstrate that Activin A signaling in the absence of E-cadherin can upregulate CD44. Furthermore, CD44 anchors MMP-9 to the invasive front translating into increased cell invasion. These results have important implications for a novel role of Activin A in the induction of EMT through upregulation of CD44.

## Methods

### Cell culture

Primary esophageal epithelial cells (keratinocytes) from normal human esophagus were established as described previously [25]. In brief, supernatants containing pFB-neo retrovirus encoding either full-length E-cadherin or a dominant-negative mutant of E-cadherin lacking the cytoplasmic tail (designated as EC) were used for transfection of hTERT-immortalized, but non-transformed, esophageal epithelial cells [28]. Empty pFBneo was used as a control. Additionally, a dominant-negative mutant of TGFβRII (a gift of Dr. H. Moses, Vanderbilt University, Nashville, TN), subcloned into pBABE puro, was used to generate ECdnT cells and empty pBABE puro as a control. Full-length E-cadherin in pFBneo was also used to restore E-cadherin expression in TE-8 and FLO-1 cells. Fetal esophageal fibroblasts were grown in DMEM with 10% fetal bovine serum (FBS, Hyclone, Thermo Fisher Scientific, Waltham, MA), 100 units/mL penicillin, and 100 µg/mL streptomycin (Gibco, Invitrogen, Carlsbad, CA). The esophageal squamous carcinoma cell lines, TE cells, were a kind gift from Drs. Rustgi and Nakagawa (University of Pennsylvania, Philadelphia, PA). Esophageal adenocarcinoma cell lines OE33, FLO-1 and SK-GT-4 [29]–[31] and the head and neck cancer cell lines JHU-012 and JHU-013 were developed from primary head and neck squamous cell carcinoma in the Division of Head and Neck Cancer Research at the Johns Hopkins University.

For E-cadherin inhibition, cells were seeded at a density of 2x 10^5^ cells per well in a 6- well plate. After 24 hours, medium was changed to serum-free DMEM and cells were transfected with a final concentration of 10 nM siRNA using calcium phosphate transfection (400 µL of RNAse-free water was mixed with 50 µL 2.5 M CaCl_2_, 50 µL of 2 µM siRNA with 500 µL of HBSP2x (280 mM NaCl, 1.5 mM NA_2_PO_4_2H_2_O, 12 mM glucose, 10 mM KCL, 50 mM HEPES pH7.05 in 500 mL RNAse-free water)). siRNA were obtained from Qiagen (Qiagen, Valencia, CA), Hs-CDH1_12 (si1) and Hs_CDH1_13 (si2) were used against E-cadherin and AllStars negative, non-silencing siRNA probes were used for control (ctrl). For stimulation, 20 ng/ml of Activin A (R&D, Minneapolis, MN) was added to the culture.

### Tissue microarrays

Esophageal squamous carcinoma tissues were procured via surgery at the Okayama University Hospital, Kitano Hospital and the Hospital of the University of Pennsylvania through the Cooperative Human Tissue Network (CHTN). All were pathologically diagnosed as esophageal squamous cell carcinoma (ESCC) [32]. Additionally, a commercial tissue array for ESCC, AccuMax Array (ISU ABXIS, Co., distributed by Accurate Chemical & Scientific Corp., Westbury, NY) that contains 80 esophageal squamous cancer formalin fixed paraffin embedded (FFPE) tissues from 40 patients and 4 normal controls was used for immunhistochemical staining. Together, data could be collected for 166 squamous tumor samples, 2 cases with the progression from normal, carcinoma *in situ* to ESCC, and additional normal tissue controls.

One-hundred-and-thirty-three esophageal adenocarcinoma (EAC) FFPE samples were analyzed after immunohistochemical staining. These samples were obtained from the archives of the departments of pathology at Vanderbilt University (Nashville, TN, USA), Otto-von-Guericke University (Magdeburg, Germany) and from the CHTN [33]. Histopathological diagnosis of Barrett's esophagus, dysplasia and esophageal adenocarcinoma was verified on the basis of H&E-stained sections according to the Vienna classification of gastrointestinal epithelial neoplasia [34]. Immunohistochemistry staining was performed using anti-pan CD44 antibody, clone F10-44-2, and anti-E-cadherin antibody, clone 36. Expression scored on a scale from 0 to 3 with 0 being absent, and 3 being the highest signal intensity, and membranous or cytoplasmic localization of staining was recorded. Samples with scores of 1 or higher and with membranous localization were considered positive. For statistical analysis positive signals regardless of the intensity were considered positive and scores below 1 as negative and statistically analyzed using 2×2 contingency tables and Fisher's exact test.

### Organotypic culture

Organotypic cultures were grown as described previously [25]. In brief, human esophageal epithelial cells (keratinocytes) were seeded onto a 3∶1 collagen I/matrigel layer with 7.5×10^4^ human fetal esophageal fibroblasts embedded. Collagen I was purchased from Organogenesis (Canton, MA) and Matrigel Matrix from BD Bioscience (Franklin Lakes, NJ). On day 11, cultures were raised to an air–liquid interface to induce differentiation of the epithelium. Cultures were harvested on day 15 and either fixed in 10% formaldehyde (Fisher, Pittsburg, PA) for paraffin embedding or directly embedded into Tissue-Tek O.C.T. compound (VWR, Batavia, IL) for frozen sections.

### Cell Invasion Assays

Invasion assays were performed as previously described [25] using 8 µm pore size Biocoat Matrigel Fluoroblok invasion chambers (BD Biosciences, Franklin Lakes, NJ). Epithelial cells (5×10^4^ per chamber) were seeded into the transwell inserts and the invading cells stained with Calcein AM (Invitrogen, Carlsbad, CA) after overnight incubation. Activin A was added with a concentration of 20 ng/ml as a chemoattractant. Invasion was quantified using the Multimode Plate Reader Synergy HT (Bio-Tek, Winooski, VT). All experiments were done in triplicate and repeated twice. Data are presented as mean±SD. Man and Whitney test was performed for statistical analysis.

### Antibodies

CD44 Hermes-III (a kind gift from Dr. Ellen Pure, Wistar Institute, Philadelphia, PA) was used for immunofluoresence on frozen sections; anti-CD44 clone 2C5 (R&D systems, Minneapolis, MN) for Western Blot; CD44 F10-44-2 and α-tubulin (Abcam, Cambridge, MA) were used for immunohistochemistry on FFPE and Western Blot, respectively; E-cadherin purchased from BD Bioscience (Franklin Lakes, NJ), β-actin and αSMA clone 1A4 were both purchased from Sigma (Saint Louis, MO), anti-MMP-9 from Calbiochem (EMD Chemicals, Rockland, MA); S100A4 is from Thermo Fisher Scientific (Fremont, CA) and vimentin clone V9 from Santa Cruz Biotechnology Inc. (Santa Cruz, CA).

### Immunohistochemistry and Immunofluorescence

Immunohistochemistry was performed with the Vecta Elite kit (Vector Laboratories, Burlingame, CA) following the manufacturer's protocol using their reagents. Primary antibody was incubated overnight at 4°C and secondary antibody for 30 minutes at 37°C. Then, the signal was developed using the DAB substrate kit for peroxidase. For immunofluoresence staining, a biotinylated secondary antibody was detected using Texas-Red-Streptavidin or FITC-label secondary antibody. Stained sections were mounted with DAPI-containing mounting medium.

### Zymography and in situ zymography

Conditioned media from organotypic culture grown under serum-free conditions for 48 hours were separated on a 10% acrylamide gel containing gelatin at 4°C. Gels were stained with Coomassie blue R250 for 2 hours and excess of coloration was removed with destain buffer (10% acetic acid 20% methanol). Images were taken using Gel Doc™ XR (Bio Rad, Hercules, CA).

For in situ zymography, DQ-Gelatin fluorescein conjugate (Molecular Probes, Invitrogen, Carlsbad, CA) was resuspended in 1 ml deionized water at a concentration of 1 mg/ml. 100 ug/ml DQ-Gelatin was used to overlay 5 micron thick frozen sections of organotypic cultures for 18 hours at 37°C. The fluorescein signal on the sections was imaged using a fluorescence microscope (Zeiss, Thornwood, NY).

### Western blotting

Western Blots were performed as described before [35]. Experiments were repeated at least twice.

## Results

### Primary human esophageal tumors show inverse expression of E-cadherin and CD44

**Figure 1:**
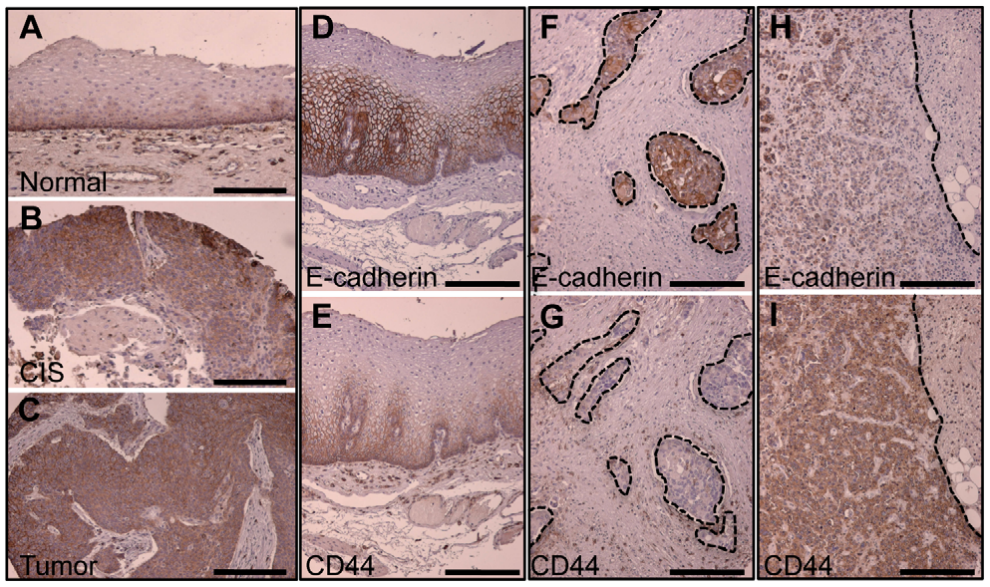
Inverse correlation of E-cadherin and CD44 expression in primary human esophageal tumors. IHC with anti-CD44 antibody shows increased CD44 expression during the progression from normal (A) to carcinoma *in situ,* CIS (B), and tumor (C). Antibody against E-cadherin and CD44 show expression of E-cadherin (D) and in the absence of CD44 (E) in the normal epithelium. In EAC tissues with retained E-cadherin expression (F, dashed black line) the signal for CD44 is low (G). (H) Loss of E-cadherin is associated with an intense signal for CD44 (I). Scale bar is 50 micron.

To determine the role of CD44 in esophageal cell invasion and EMT, we performed immunohistochemical staining of E-cadherin and CD44 on tissue microarrays of 166 squamous cell carcinoma (SCC) and 131 adenocarcinomas. For two esophageal SCC patients, samples representing the progression from normal to carcinoma *in situ* (CIS) and subsequently ESCC (Fig. 1A to 1C), we observed a gradual increase of CD44 expression. Overall, E-cadherin was largely restricted to areas with low or absent CD44 expression in the normal epithelium (Fig. 1D, E) and 20 out of 166 tumors. For esophageal SCC, we observed upregulation of CD44 in the absence of E-cadherin in 54% of the tumors (90 out of 166 tumors). Together, 110 out of 166 (66%, p<0.02) of our clinical samples displayed this inverse relationship of E-cadherin and CD44. Esophageal adenocarcinoma samples showed an inverse correlation of E-cadherin and CD44 expression in 63 out of 131 samples (48%), with 41 samples having substantial E-cadherin, but no CD44 expression. Twenty-two samples had increased CD44 expression and are negative for E-cadherin. Taken together, we show a statistical significant (p<0.05) inverse correlation for the expression of E-cadherin and CD44. Images of representative immunohistochemical stainings of tumor tissues demonstrating the association of E-cadherin and CD44 are shown in Fig. 1 F-I.

### E-cadherin-negative esophageal cancer cell lines show increased CD44 expression

**Figure 2:**
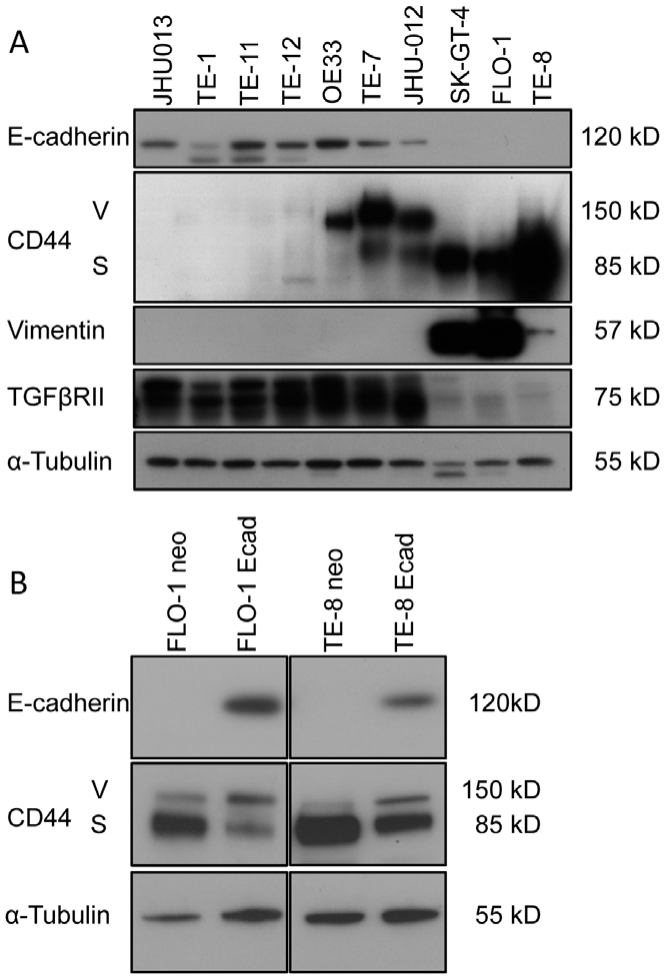
Expression of CD44, TGFβRII, vimentin and E-cadherin in cancer cell lines. (A) Western Blot analysis of esophageal squamous carcinoma cell lines (TE-1 -7, -8, -11, and -12), esophageal adenocarcinoma cell lines (FLO-1, OE33, SK-GT-4) and head and neck cancer cell lines (JHU-012, JHU-013) show that expression of E-cadherin and TGFβRII is associated with low levels of CD44, while loss of E-cadherin and TGFβRII correlates with up-regulation of CD44 and the EMT marker vimentin. (B) Restoration of E-cadherin, Ecad, expression by retroviral transfection of TE-8 and FLO-1 cells compared to vector control (neo) demonstrates an overall decrease in CD44 expression along with a switch to increased CD44v over CD44s (CD44v: CD44variant, CD44s: CD44 standard).

To investigate the inverse correlation of E-cadherin with CD44 in esophageal cancer cell lines, we analyzed esophageal squamous cell carcinoma cell lines (TE-1, -7, -8, -11 and -12), head-and-neck squamous cancer cell lines (JHU-012, JHU-013), as well as adenocarcinoma cell lines (FLO-1, OE33 and SK-GT-4) by Western Blot for E-cadherin and CD44 expression as well as TGFβRII and vimentin. While the standard form of CD44 (CD44s) is expressed in mesenchymal cells, some variant forms, CD44v, are expressed in epithelial cells [36]. We found that cells retaining E-cadherin expression had lower levels of standard (85 kDa due to the lack of exon 6–15) and the variant form (150 kDa) of CD44. The loss of E-cadherin correlates with the increased expression of CD44s and an increase in expression of the EMT marker vimentin (Fig. 2A). To determine if restoration of E-cadherin can affect the expression of CD44s and CD44v, we transfected TE-8 and FLO-1 cell lines, both express low levels of E-cadherin, with full-length E-cadherin. Upon restoration of E-cadherin expression, the overall level of CD44 expression was decreased, but the variant (epithelial) form (125 kDa) increased (Fig. 2B).

### CD44 expression is upregulated in esophageal epithelial cells undergoing EMT

**Figure 3:**
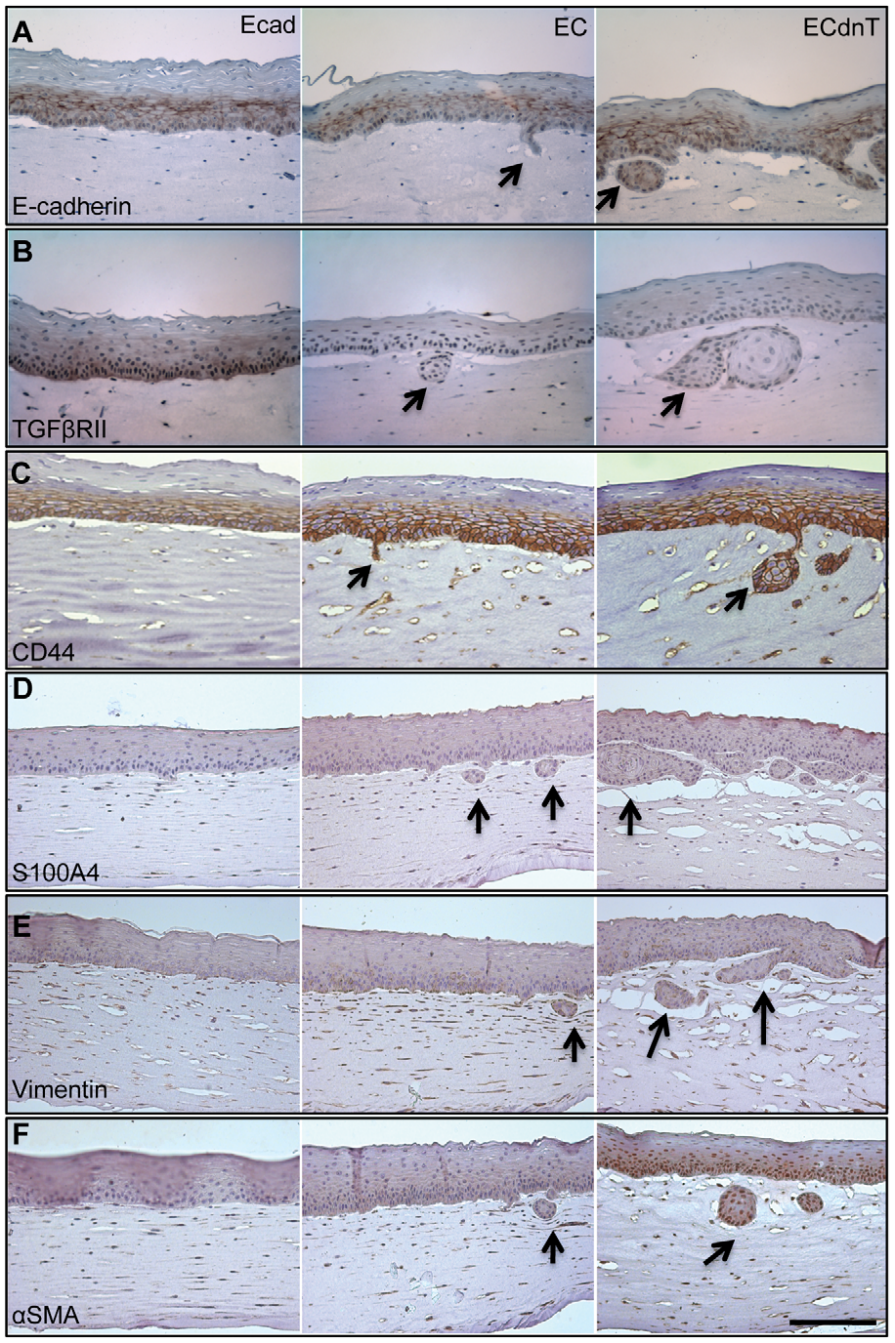
CD44 expression is increased in areas of invasion after E-cadherin loss. Immunohistochemistry of paraffin sections with anti-E-cadherin antibody (A), anti-TGFβRII (B) and anti-CD44 (C) shows CD44–positive cells in EC and ECdnT cells invading into the underlying collagen/matrigel matrix (arrows) that are negative for E-cadherin and TGFβRII. Immunohistochemical staining for the EMT markers S100A4 (D), vimentin (E) and αSMA (F) shows increased positive signal in invading cells. Scale bar is 50 micron.

We previously established a model to analyze the effects of E-cadherin loss on esophageal keratinocytes by retroviral expression of dominant-negative E-cadherin (EC) or dominant-negative E-cadherin in combination with dominant-negative TGFβRII (ECdnT) [25]. We performed immunohistochemical stainings with antibodies against E-cadherin and CD44 and markers of EMT to elucidate the role of CD44 in esophageal EMT and cell invasion. EC and ECdnT cells have upregulated CD44 expression, and the positive CD44 signal largely localizes to areas of epithelial cell invasion into the underlying matrix (Fig. 3). Both EC and ECdnT cells have increased levels of S100A4, vimentin and αSMA indicating that these cells are undergoing EMT in organotypic cultures (Fig. 3D–F).

### CD44 co-localizes with MMP-9 at the leading edge of invasive areas and is upregulated by Activin A

**Figure 4:**
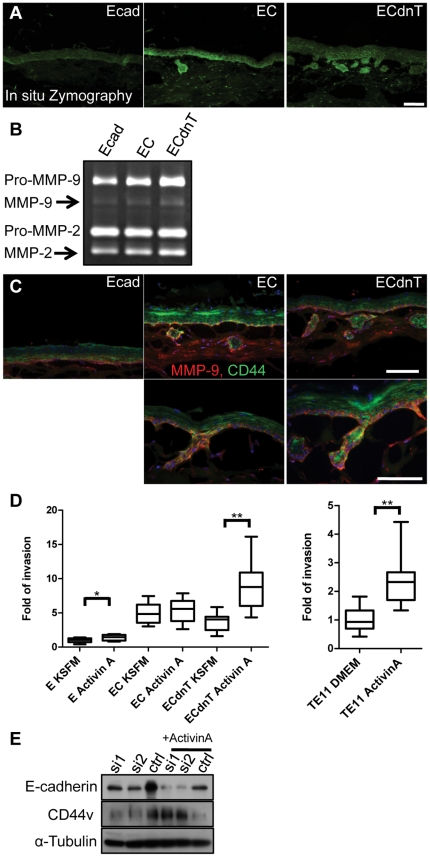
CD44 co-localizes with MMP-9 in areas of invasion and is upregulated by Activin A. (A) For *in situ* zymography, areas of MMP activity are highlighted by a positive fluorescein signal on frozen sections of Ecad, EC and ECdnT organotypic cultures. (B) Zymography using organotypic culture conditioned medium collected from Ecad, EC and ECdnT cells illustrates increased secretion of MMP-2 and MMP-9. (C) Double immunofluorescence staining of Ecad, EC and ECdnT shows MMP-9 (red) colocalization with CD44 (green) in invasive cells. Scale bar is 50 micron. (D) Stimulation with Activin A induces enhanced invasion of ECdnT and TE-11 cells in invasion assays. Comparison between groups was done by Man and Whitney non- parametrical test, * p<0.05, **p<0.001. (E) Western Blot of cell lysates from TE-11 transfected with AllStars negative non-silencing siRNA cells (ctrl), and TE-11 cells transfected with siRNA against E-cadherin (si1, si2) demonstrates upregulation of CD44 after stimulation with Activin A.

To analyze the mechanism by which CD44 mediates cell invasion we focused on the activation of matrix metalloproteinases, prominent proteases digesting the extracellular matrix. To visualize MMP activity in areas of invasion we used in situ zymography. Fluorescein-positive areas detecting MMP activity were restricted to the leading edge in EC and ECdnT cells and cell invasion into the underlying matrix (Fig. 4A). Conditioned media from organotypic cultures showed a gradual increase of MMP-9 secretion and MMP-2 and MMP-9 activity in the invasive cells (Fig. 4B). MMP-9 has been described as a binding partner of CD44 [37], [38]. Using double immunofluoresence staining with antibody against CD44 and MMP-9 we illustrate their co-localization at the cell surface on the leading edge of invasive areas (Fig. 4C). To demonstrate an increased invasive potential in response to Activin A, a TGFβ family member, secretion of which is increased in the invasive organotypic cultures (data not shown), we stimulated ECdnT cells and the esophageal cancer cell line TE-11 and show enhanced cell invasion *in vitro* (Fig. 4D).

Activin A has also been described to regulate stemness [17] and we hypothesized that CD44 as a stem cell marker might be under the regulation of Activin A. To identify a potential signaling pathway responsible for the upregulation of CD44, we analyzed the esophageal cancer cell line, TE-11, after knockdown of E-cadherin using siRNA. We stimulated TE-11 cells transfected with siRNA against E-cadherin cells with Activin A and analyzed the expression of CD44. Activin A treatment increased CD44 variant expression in TE-11 in the absence of E-cadherin (Fig. 4E).

Taken together, these data demonstrate the regulation of CD44 expression by E-cadherin through Activin A and provide functional evidence for a causal role in the induction of cell invasion.

## Discussion

We show the gradual loss of E-cadherin is associated with a progressive increase of CD44 *in vitro* and *in vivo*. In the absence of E-cadherin, Activin A can induce CD44 expression. Activin A was shown to be a key regulator for stemness [17] and like other members of the TGFβ superfamily, has also been reported to affect development and tumorigenesis [20], [22]–[24]. Overexpression of Activin A has been described in esophageal squamous cell carcinoma and is associated with poor prognosis. Activin A promotes tumor cell aggressiveness by increased expression of MMP-7 and N-cadherin [22]–[24]. Here we show that Activin A can upregulate CD44, which in turn anchors MMP-9 to the leading edge and induces cell invasion. The association of E-cadherin, CD44 and MMPs with EMT suggests a major role for Activin A in EMT.

We found that E-cadherin loss is accompanied by a change of CD44 isoform expression. Although previous studies have shown that alternative splicing of CD44 could be controlled through ras signaling to promote expression of variant forms [39], Warzecha et al. have recently identified epithelial splicing factors (ESRP1 and ESRP2) promoting the expression of CD44v to maintain the epithelial phenotype [40]. Those factors are lost during EMT [41]–[43]. Conceivably, expression of standard CD44 is dependent on loss of ESRP1 and 2 in esophageal carcinoma. ESRP1 and 2 expression has been further recognized to be implicated in EMT-associated splicing programs in breast cancer [44]. Interestingly, silencing of ESPR1/2 in epithelial cells induced expression of the mesenchymal cadherin, N-cadherin, without changes in E-cadherin expression. Conversely, expression of ESPR1 can reverse Twist-induced EMT in mammary epithelial cells. In addition, the transcription factor Twist2 contributes to the expansion of stem-like cell populations through downregulation of E-cadherin and increased expression of stem cell markers such as CD44 [45].

Taken together, there are multiple mechanisms by which CD44 isoforms, all share the same cytoplasmic domain, can induce EMT, many of which involve the interaction of the extracellular domain with the microenvironment. CD44 can act as a co-receptor for c-Met and is the bona fide receptor for hyaluronic acid, which can promote EMT [9]. E-cadherin negatively regulates the interaction of CD44 with hyaluronic acid resulting in a suppression of tumor invasion and cell branching morphogenesis [46]. We show here that the loss of E-cadherin expression correlates with the CD44s form and induction of an invasive phenotype.

We have previously shown that the invasion of esophageal keratinocytes lacking functional E-cadherin and TGFβRII was mediated by cathepsin B [35]. Cathepsin B can be activated in an acidic environment initiated by the interaction of CD44 with a Na+-H+ exchanger [37]. While increased cathepsin B activity results in TGFβ activation [35], MMP-9 can also activate TGFβ [47] linking the data presented here with our previous study. Paracrine TGFβ signaling activates fibroblasts, and also induces capillary formation [48]. These observations highlight the importance of the epithelial-mesenchymal crosstalk in tumorigenesis.

In conclusion, we show that CD44 upregulation is associated with the loss of E-cadherin in esophageal carcinoma and that CD44 promotes MMP-9 mediated tumor invasion.
